# Personal and Environmental Risk Factors at Birth and Hospital Admission: Direct and Vitamin D-Mediated Effects on Bronchiolitis Hospitalization in Italian Children

**DOI:** 10.3390/ijerph18020747

**Published:** 2021-01-17

**Authors:** Marco Zaffanello, Giuliana Ferrante, Salvatore Fasola, Michele Piazza, Giorgio Piacentini, Stefania La Grutta

**Affiliations:** 1 Department of Surgery, Dentistry, Paediatrics and Gynaecology, University of Verona, 37126 Verona, Italy; marco.zaffanello@univr.it (M.Z.); michele.piazza@univr.it (M.P.); giorgio.piacentini@univr.it (G.P.); 2 Department of Health Promotion, Mother and Child Care, Internal Medicine and Medical Specialties, University of Palermo, 90127 Palermo, Italy; giuliana.ferrante@unipa.it; 3 Institute for Biomedical Research and Innovation, National Research Council, 90146 Palermo, Italy; stefania.lagrutta@irib.cnr.it

**Keywords:** bronchiolitis hospitalization, gestational age, mediation analysis, seasonality, vitamin D

## Abstract

Seasonal variations in UV-B radiation may influence vitamin D status, and this, in turn, may influence the risk of bronchiolitis hospitalization. The aim of this study was using a causal inference approach to investigate, simultaneously, the interrelationships between personal and environmental risk factors at birth/hospital admission (RFBH), serum vitamin D levels and bronchiolitis hospitalization. A total of 63 children (<2 years old) hospitalized for bronchiolitis (34 RSV-positive) and 63 controls were consecutively enrolled (2014–2016). Vitamin D levels and some RFBH (birth season, birth weight, gestational age, gender, age, weight, hospitalization season) were recorded. The discovered RFBH effects on the risk ok bronchiolitis hospitalization were decomposed into direct and vitamin-D mediated ones through Mediation Analysis. Winter-spring season (vs. summer-autumn) was significantly associated with lower vitamin D levels (mean difference −11.14 nmol/L). Increasing serum vitamin D levels were significantly associated with a lower risk of bronchiolitis hospitalization (OR = 0.84 for a 10-nmol/L increase). Winter-spring season and gestational age (one-week increase) were significantly and directly associated with bronchiolitis hospitalization (OR = 6.37 and OR = 0.78 respectively), while vitamin D-mediated effects were negligible (1.21 and 1.02 respectively). Using a comprehensive causal approach may enhance the understanding of the complex interrelationships among RFBH, vitamin D and bronchiolitis hospitalization.

## 1. Introduction

Bronchiolitis is one of the most common lower respiratory tract infections in children younger than two years of age [1]. Lower gestational age (GA) is one of the main host risk factors for bronchiolitis requiring hospitalization during the epidemic seasons [2]. Even early-term birth (37 + 0–38 + 6 GA) has been associated with an increased risk of admission for bronchiolitis [3].

The role of vitamin D in bronchiolitis is still controversial. A deficit of vitamin D serum levels has been described in children aged <2 years hospitalized for bronchiolitis [4]. Conversely, Roth et al., in a study on children aged 1–25 months admitted to hospital with lower respiratory tract infections (primarily viral bronchiolitis), found that serum vitamin D levels were not associated with the risk of hospitalization [5].

The seasonality of bronchiolitis is an area of expanding research [6]. A previous study at the population level reported reduced vitamin D serum concentrations during winter, possibly due to reduced ultraviolet-B (UV-B) radiation exposure [7]. A nationwide ecological study found an inverse correlation between regional solar radiation as a proxy of vitamin D status and incidence of hospital admissions for bronchiolitis in Chile [8]. Seasonal variations in UV-B radiation cause seasonal variations in vitamin D status and this may account for increased susceptibility to infectious respiratory diseases [9]. Therefore, it could be speculated that winter vitamin D reduction may play a role in bronchiolitis.

To date, no study has investigated simultaneously the interrelationships between personal and environmental risk factors at birth and hospital admission (RFBH), serum vitamin D levels and bronchiolitis hospitalization in children using causal inference approaches. In this study, we aimed to use Mediation Analysis (MA) to discover whether, and the extent to which, the link between RFBH and bronchiolitis hospitalization could be ascribed to a direct or a vitamin-D mediated (indirect) effect.

## 2. Materials and Methods

### 2.1. Study Design and Population

**Figure 1:**
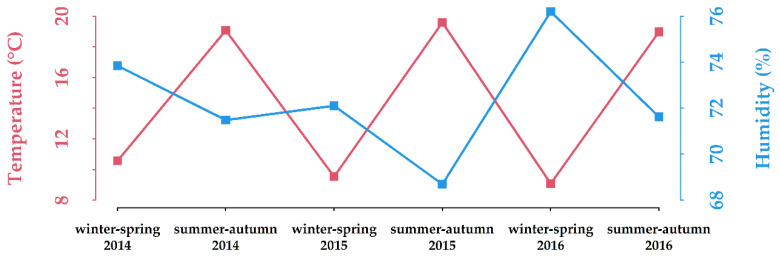
Seasonal means of temperatures and relative humidity recorded in Verona (Villafranca monitoring station, VILSPA; www.ilmeteo.it/portale/archivio-meteo/Verona) through the study period. Winter-spring: 1 December to 31 May; summer-autumn: 1 June to 30 November.

This case-control study was carried out in Caucasian infants consecutively hospitalized between 1 January 2014, and 30 November 2016, at the Pediatric Section of the Department of Surgery, Dentistry, Pediatrics and Gynecology, University of Verona, Italy. Geographic coordinates for Verona are 45°26′19″ N 10°59′34″ E. Seasonal means of temperatures and relative humidity recorded in Verona through the study period are represented in Figure 1.

The study was approved by the local Ethics Committee for Clinical Trials (approval number CESC 0173, 2012) and was performed according to the good clinical practice principles set out in the Declaration of Helsinki. On enrolment, all the parents/legal guardians gave informed consent to participate in the study.

A group of 126 hospitalized children below two years of age, 63 with bronchiolitis (cases) and 63 controls matched by age and gender were consecutively enrolled. We identified children admitted with a primary diagnosis of acute bronchiolitis using the “J21” ICD-10 codes (“J210”—acute bronchiolitis due to respiratory syncytial virus—RSV; “J218”—acute bronchiolitis due to other specified organisms; “J219”—acute bronchiolitis, unspecified). The diagnosis of viral bronchiolitis was carried out by genome detection using polymerase chain reaction (PCR) assay on nasal lavage drawn from a nasopharyngeal aspirate having good sensitivity for detection of RSV [10]. Bronchiolitis was classified as RSV-positive (+) or RSV-negative (−). Children admitted for non-infectious diseases (i.e., angiomas, traumas, foreign bodies, accidental ingestion of drugs) served as controls. All the children who required admission in the pediatric intensive care unit, and those presenting complex comorbidities (e.g., chronic lung disease or congenital abnormalities) were excluded.

### 2.2. Procedures

Gender, age, weight and hospitalization seasons, categorized as winter-spring (from 1 December to 31 May) and summer-autumn (from 1 June to 30 November) [11], were recorded. In addition, gestational age, birth weight and birth season (same categorization) were retrospectively recorded. Serum vitamin D levels were measured at hospital admission and were recorded as absolute concentrations (nmol/L). Serum vitamin D levels were measured by a chemiluminescent assay (DiaSorin LIAISON automated immunoassay analyzer) and expressed as ng/mL. The conversion factor used to transform vitamin D ng/mL into nmol/L is 2.496 (nmol/L = 2.496 × ng/mL).

### 2.3. Statistical Analysis

Vitamin D levels and RFBH were compared in cases and controls through a *t*-test for two means (quantitative variables) and chi-square test for percentages (categorical variables).

**Figure 2:**
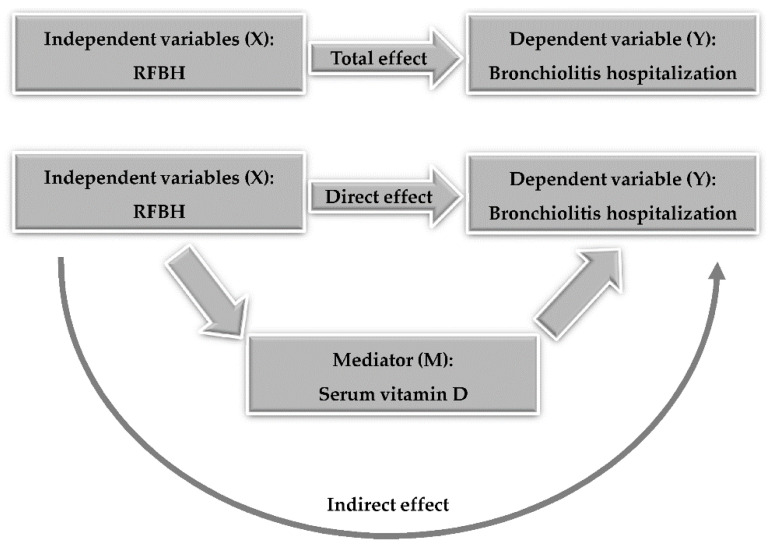
Mediation Analysis. Some X-Y association, that is, the “total effect”, is initially assessed. Then, a mediator M is introduced, and the aforementioned association may decrease, increase, or remain the same; this is referred to as the “direct effect”. The difference between the total and the direct effect is therefore to ascribe to an “indirect effect” of X on Y via M. RFBH: risk factors at birth and hospital admission.

The extent to which the relationship between the RFBH and bronchiolitis hospitalization could be ascribed to a direct or vitamin D-mediated (indirect) effect was investigated through a MA. In MA, some X-Y (RFBH-bronchiolitis hospitalization) association, that is, the “total effect”, is initially assessed; then, a mediator M (vitamin D) is introduced, and the X-Y association may decrease, increase, or remain the same; this is referred to as the “direct effect”. The difference between the total and the direct effect is therefore the “indirect effect” of X on Y via M (Figure 2).

A preliminary analysis was carried out to detect the RFBH potentially associated (either directly or indirectly) with bronchiolitis hospitalization. In particular, a linear regression model with serum vitamin D level (the mediator) as the response variable, and the RFBH as independent variables, was estimated to detect the RFBH that might, potentially, influence the risk of bronchiolitis via the mediator. Similarly, a logistic regression model with bronchiolitis hospitalization as the response variable (yes or no), and the RFBH as independent variables, was estimated to detect the RFBH with a non-null total effect on the risk of bronchiolitis. For both models, a backward stepwise variable selection procedure was performed, using the Akaike Information Criterion (AIC) as an optimality criterion.

The selected RFBH were included in the MA, and their effects were decomposed into direct and indirect ones (mediated by serum vitamin D levels). Since structural equation modeling cannot be used for binary outcomes, the decomposition method described by Karlson–Holm–Breen (KHB) was applied [12], using the implementation in the *khb* library [13] of the *R* statistical software. For the final bronchiolitis model (including vitamin D and the selected RFBH), the area under the receiver operating characteristic (ROC) curve (AUC) was also reported.

All the statistical analyses were performed through R version 4.0.2 (R Foundation for Statistical Computing, Vienna, Austria); *p*-values lower than 0.05 were considered statistically significant. MA results were expressed as β coefficients (mean differences, MD) for the linear regression model (vitamin D), and odds ratios (OR) with 95% confidence intervals (CIs) for the logistic regression model (bronchiolitis hospitalization).

## 3. Results

A total of 63 children hospitalized for bronchiolitis (34 RSV+ and 29 RSV−) and 63 controls participated in the study. Table 1 summarizes the characteristics of the study sample. Overall, the mean age at hospitalization was about 6 months; 61 children (48%) were males and 65 (52%) were females. Age and gender distribution were similar between cases and controls. Cases were more frequently hospitalized in winter-spring than controls (83% vs. 46%, *p* < 0.001). Mean gestational age was significantly lower in cases than in controls (38.1 vs. 39.1 weeks, *p* = 0.030), and a higher percentage of cases were born preterm (<37 weeks, 18% vs. 2%, *p* = 0.006). Mean serum vitamin D level (range 27.4–170.2 of nmol/L) was significantly lower in cases than in controls (66.4 vs. 78.6 nmol/L, *p* = 0.012), even if the percentage of children with vitamin D deficiency-insufficiency (<75 nmol/L) [14] was not significantly different (64% vs. 52%, *p* = 0.279).

The hospitalization season was the only variable selected by the stepwise procedure in the preliminary linear model for vitamin D, while the hospitalization season and the gestational age were the variables selected in the preliminary logistic regression model for bronchiolitis hospitalization; therefore, these variables were further investigated in the MA.

**Figure 3:**
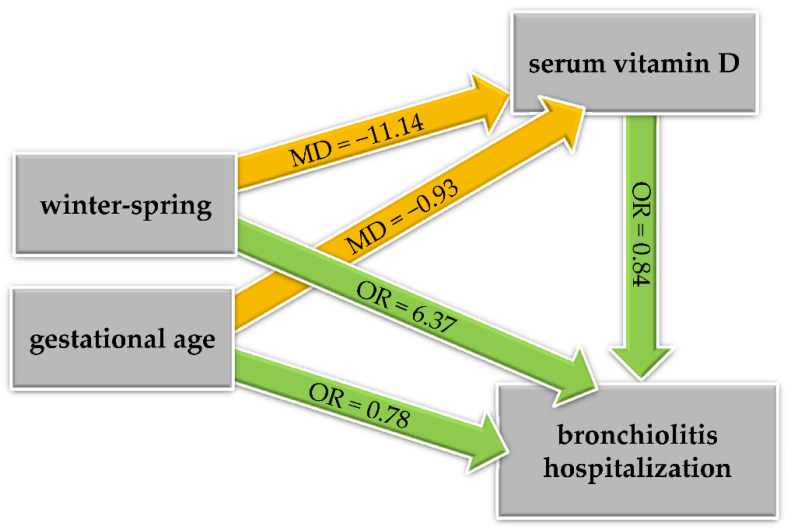
Directed acyclic graph illustrating the mediation analysis. MD: mean difference (orange); OR: odds ratio (green).

Table 2 and Figure 3 summarize the results of the final models, that is, the linear regression model for vitamin D (mediator), and the logistic regression model for the risk of bronchiolitis hospitalization, along with the KHB effect decomposition.

**Figure 4:**
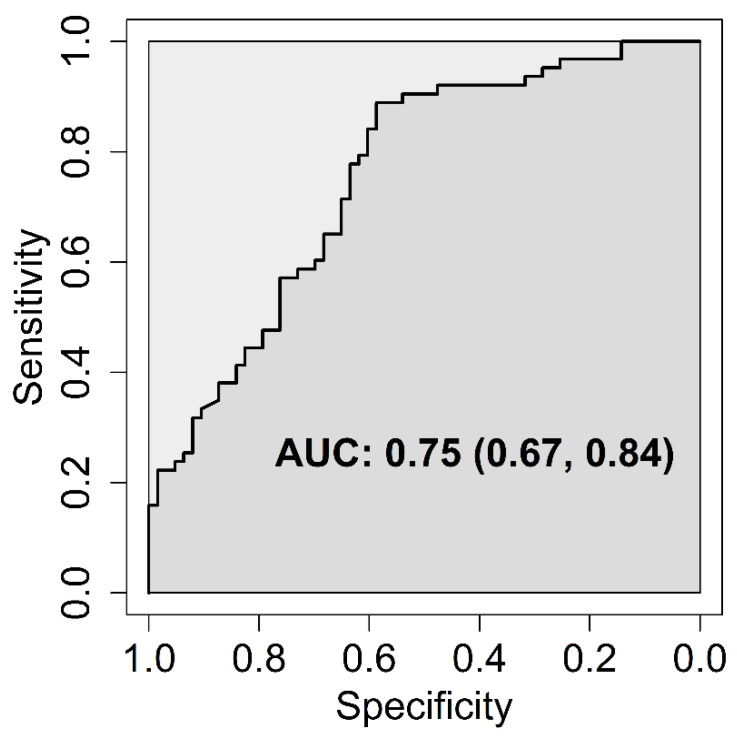
Receiver operating characteristic (ROC) curve. Outcome: bronchiolitis hospitalization; predictor: fitted probability from the logistic regression model.

Increasing serum vitamin D levels were significantly associated with a lower risk of bronchiolitis hospitalization (OR = 0.84, 95% CI: [0.72, 0.98] for a 10-nmol/L increase, *p* = 0.035). Being hospitalized in winter-spring was significantly associated with a lower serum vitamin D level (MD = −11.14 nmol/L, 95% CI: [−21.05, −1.23], *p* = 0.029) and a higher risk of bronchiolitis hospitalization, for which the effect was predominantly direct (OR = 6.37, 95% CI: [2.58, 15.71], *p* < 0.001); indeed, only 9% of the total effect was mediated by the serum vitamin D level. An increased gestational age was significantly and directly associated with a lower risk of bronchiolitis hospitalization (OR = 0.78, 95% CI: [0.65, 0.94] for a one-week increase, *p* = 0.008), and the direct effect was slightly larger than the total effect (OR = 0.79, 95% CI: [0.66, 0.95], *p* = 0.012); indeed, the direction of the indirect effect was opposite (OR = 1.02, 95% CI: [0.98, 1.05], *p* = 0.334). This can be referred to as a case of “inconsistent mediation” or “suppression effect” and the percent mediated effect is meaningless in this context [15]. Indeed, increasing gestational age was tendentially associated with a lower vitamin D level (MD = −0.93 nmol/L, 95% CI: [−2.63, 0.77], *p* = 0.285), which was in turn associated with a higher risk of bronchiolitis hospitalization. The AUC associated with the logistic regression model for bronchiolitis hospitalization was 0.75 (95% CI: 0.67–0.84); the ROC curve is depicted in Figure 4.

## 4. Discussion

Mediation analysis showed that the winter-spring season was associated with low serum vitamin D levels, which were in turn associated with an increased risk of bronchiolitis hospitalization in children aged <2 years; despite that, the path of association between the winter-spring season and bronchiolitis hospitalization was found to be predominantly direct rather than vitamin D-mediated. A lower gestational age was also found to be directly associated with bronchiolitis hospitalization.

We found lower serum vitamin D levels in children hospitalized in winter-spring. Previous studies on the correlation between seasonality and vitamin D levels in children below two years of age have shown conflicting results [16]. The current study results are in agreement with a previous study on 353 New Zealand infants aged ≤2 years, showing an increased risk of vitamin D deficiency associated with measurement in winter or spring (RR = 7.24, 95% CI 1.55, 23.58) [17]. More recently, a study on Italian children showed a significantly increased risk of hypovitaminosis D in blood samples taken in winter (OR = 27.20) and spring (OR = 26.44) compared to summer [18]. Overall, our results seem to confirm the association between seasonality and vitamin D levels.

We found that low serum vitamin D levels were associated with an increased risk of bronchiolitis hospitalization. So far, conflicting results have been reported about this association. Our finding is in line with a study on 102 children aged 1–24 months with bronchiolitis, in which vitamin D concentration was significantly lower in the hospitalized group than in the non-hospitalized group [19]. Moreover, a deficit of vitamin D serum levels has been described in children aged <2 years hospitalized for bronchiolitis in the previous study by Halasa et al. [4]. Conversely, a study on children aged 1–25 months admitted to hospital with lower respiratory tract infections (primarily viral bronchiolitis), did not find any association between serum vitamin D levels and the risk of hospitalization [5]. At last, no significant difference between children aged 1–24 months hospitalized with acute bronchiolitis and controls was highlighted in a recent case-control study [20].

In the current study, the path of association between the season and bronchiolitis hospitalization was found to be predominantly direct rather than vitamin D-mediated. In regions with a temperate climate, bronchiolitis generally occurs in annual outbreaks starting towards the end of autumn and lasting until spring [21]. In the Netherlands, during the period 1998–2005, relative humidity was positively associated with RSV activity for up to four weeks, suggesting more RSV activity when relative humidity increased and, therefore, supporting the role of climatic conditions in the dynamics of RSV infection [22]. Accordingly, in a study conducted in an urban area in Italy, the number of RSV-positive infants was significantly and positively correlated with relative humidity and negatively correlated with temperature [23].

A very recent study performed in Tunisia on children ≤5 years old hospitalized for RSV bronchiolitis reported that the epidemic season started in October and peaked in January [24]. In a cross-sectional study performed in Turkey on children younger than two years of age and hospitalized for acute viral bronchiolitis, most of the admissions were observed in January, February and March [25]. An important seasonality was observed also in a Spanish study on children up to five years of age hospitalized due to RSV infection, with 76% of the admissions occurring between November and March [26]. Whereas the possible mediating role of vitamin D was not investigated in the aforementioned studies, the current MA showed that seasonality of bronchiolitis hospitalization is not to be ascribed to an indirect effect of reduced vitamin D levels during winter-spring (vitamin D accounted for only 9% of the total effect), but rather to seasonality. Therefore, our results are in line with previous findings. Nonetheless, we observed that that increasing serum vitamin D levels were significantly associated with a lower risk of bronchiolitis hospitalization.

As expected, a lower gestational age (one-week decrease) was found directly associated with an increased risk of bronchiolitis hospitalization. Particularly, our findings are in line with the cohort study by Haataja et al., reporting an increased risk of admission for bronchiolitis in early-term birth (37 + 0–38 + 6 GA) [3]. Conversely, a large birth cohort study did not find associations between early term birth and hospital admissions for asthma, bronchitis and bronchiolitis [27]. We also found that a lower gestational age was tendentially (although not significantly) associated with higher serum vitamin D levels at hospitalization. However, it should be noted that such a weak indirect (vitamin D-mediated) effect (OR = 1.02) was not able to counteract the direct effect of gestational age on the risk of bronchiolitis hospitalization.

The main strength of this study is to have used a comprehensive causal inference approach (MA) in which the estimation of two simultaneous model equations (for vitamin D levels and for bronchiolitis hospitalization) allowed the investigation of the interrelationships among RFBH, vitamin D and bronchiolitis hospitalization, disentangling direct and indirect relationships. Moreover, routinely assessed variables were included in the analyses (there were no missing values), and this makes the results easy to interpret from an integrated health-environment perspective.

A possible study limitation is the lack of data about other potential RFBH, such as vitamin D supplementation in pregnancy, mode of delivery, feeding modality (breast/artificial feeding and its duration) and passive smoke exposure. Moreover, as with any retrospective study, the temporality of the observed associations could not be ascertained, and we cannot thus exclude “reverse causation” between low vitamin D levels and bronchiolitis hospitalization. However, to our knowledge, no previous study has provided evidence about such an inverse relationship in children so far. Vitamin D is thought to play a relevant role in reducing inflammation and in improving immune function; indeed, it has been reported to mediate the innate and adaptive immune responses and trigger effective antimicrobial pathways against pathogens [28]. Thus, future larger-scale and longitudinal studies (also in other environmental contexts) would be useful to confirm the current study findings.

## 5. Conclusions

Decreased vitamin D levels were found to increase the risk of bronchiolitis hospitalization in children aged <2 years. Although vitamin D levels were decreased during winter-spring, the association between season and bronchiolitis hospitalization was predominantly direct rather than vitamin D-mediated. Using a comprehensive causal approach may enhance the understanding of the complex interrelationships among personal and environmental risk factors, vitamin D and bronchiolitis hospitalization. Increasing evidence supports the role of climatic factors on respiratory infection morbidities in children. This advocates for pediatricians’ increased awareness and a better understanding of the environmental impact on children’s respiratory health [29].

## Figures and Tables

**Table 1:** Characteristics of the study sample by group.

	Controls *n* = 63 (50%)	Cases *n* = 63 (50%)	Overall *n* = 126 (100%)	*p*-Value ^1^
Gender, *n* (%)				0.721
Male	32 (51)	29 (46)	61 (48)	
Female	31 (49)	34 (54)	65 (52)	
Age at hospitalization (months), mean (SD)	5.9 (4.3)	6.0 (5.3)	5.9 (4.8)	0.925
Weight at hospitalization (kg), mean (SD)	7.1 (2.2)	6.5 (2.2)	6.8 (2.2)	0.185
Hospitalization season, *n* (%)				**<0.001**
Summer-Autumn	34 (54)	11 (17)	45 (35.7)	
Winter-Spring	29 (46)	52 (83)	81 (64.3)	
Birth season, *n* (%)				
Summer-Autumn	31 (49.2)	34 (54.0)	65 (51.6)	0.721
Winter-Spring	32 (50.8)	29 (46.0)	61 (48.4)	
Birth weight (kg), mean (SD)	3.2 (519.1)	3.0 (766.3)	3.1 (0.7)	0.125
Gestational age (weeks), mean (SD)	39.1 (1.6)	38.1 (3.6)	38.6 (2.8)	**0.030**
Preterm birth (<37 weeks), *n* (%)	1 (2)	11 (18)	12 (10)	**0.006**
Serum vitamin D (nmol/L), mean (SD) ^1^	78.6 (31.9)	66.4 (21.0)	72.5 (27.6)	**0.012**
Vitamin D deficiency-insufficiency (<75 nmol/L), *n* (%)	33 (52)	40 (64)	73 (58)	0.279

^1^ At hospital admission. Significant *p*-values are in bold.

**Table 2:** Characteristics of the study sample by group.

	Vitamin D MD (95% CI)	Bronchiolitis OR (95% CI)
Hospitalized in Winter-Spring		
Total effect	**−11.14 (−21.05, −1.23), *p* = 0.029**	**7.72 (3.09, 19.29), *p* < 0.001**
Direct effect	**-**	**6.37 (2.58, 15.71), *p* < 0.001**
Indirect (vitamin D-mediated) effect	-	1.21 (0.95, 1.55), *p* = 0.125
Percent vitamin D-mediated	-	9%
Gestational age (weeks), unit increase		
Total effect	−0.93 (−2.63, 0.77), *p* = 0.285	**0.79 (0.66, 0.95), *p* = 0.012**
Direct effect	-	**0.78 (0.65, 0.94), *p* = 0.008**
Indirect (vitamin D-mediated) effect	-	1.02 (0.98, 1.05), *p* = 0.334
Percent vitamin D-mediated	-	- ^1^
Vitamin D (nmol/L), 10-unit increase	-	**0.84 (0.72, 0.98), *p* = 0.035**

MD: mean difference; OR: odds ratio; CI: confidence interval. ^1^ Inconsistent mediation. Significant *p*-values are in bold.

## Data Availability

The data are not publicly available due to ethical restrictions.
